# Protocol for sample preparation and single-molecule optical tweezers study of nanodisc-embedded ABC transporter OpuA

**DOI:** 10.1016/j.xpro.2025.103869

**Published:** 2025-06-02

**Authors:** Lyan van der Sleen, Marco van den Noort, Laura-Marie Silbermann, Bert Poolman, Katarzyna Tych

**Affiliations:** 1 Department of Biochemistry Groningen Biomolecular Sciences and Biotechnology Institute, University of Groningen, Nijenborgh 3, 9747 AG Groningen, the Netherlands; 2 Chemical Biology Group, Groningen Biomolecular Sciences and Biotechnology Institute, University of Groningen, Nijenborgh 7, 9747 AG Groningen, the Netherlands

**Keywords:** Biophysics, Single-molecule assays, Protein biochemistry

## Abstract

OpuA is an osmoregulatory ATP-binding cassette transporter that undergoes different conformations upon varying salt concentrations and ligand conditions. Here, we present a protocol to study unfolding and interdomain interactions of OpuA using single-molecule optical tweezers (smOT). We describe steps for expression and purification of the protein, lipid nanodisc reconstitution, and sample preparation. We then detail procedures for smOT experiments and data analysis. This protocol also has potential application in the study of interdomain interactions and unfolding of (membrane) proteins in general.

For complete details on the use and execution of this protocol, please refer to van der Sleen et al.^1^

## Highlights

• Instructions for sample preparation of a membrane protein for optical tweezers
• Guidance on measuring unfolding and interdomain interactions using optical tweezers
• Steps to analyze and interpret optical tweezers data on unfolding and interactions

## Abstract (editor highlights)

Publisher’s note: Undertaking any experimental protocol requires adherence to local institutional guidelines for laboratory safety and ethics.

## Graphical abstract

**Figure:**
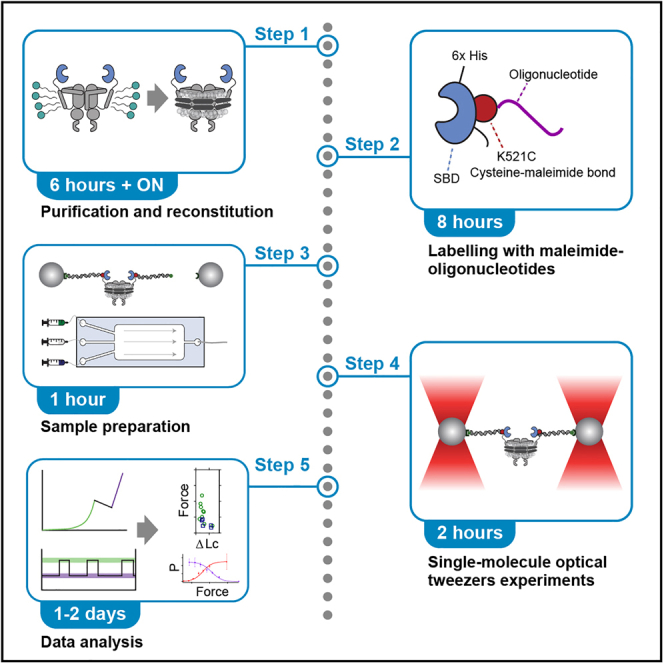

## Before you begin

**Figure 1:**
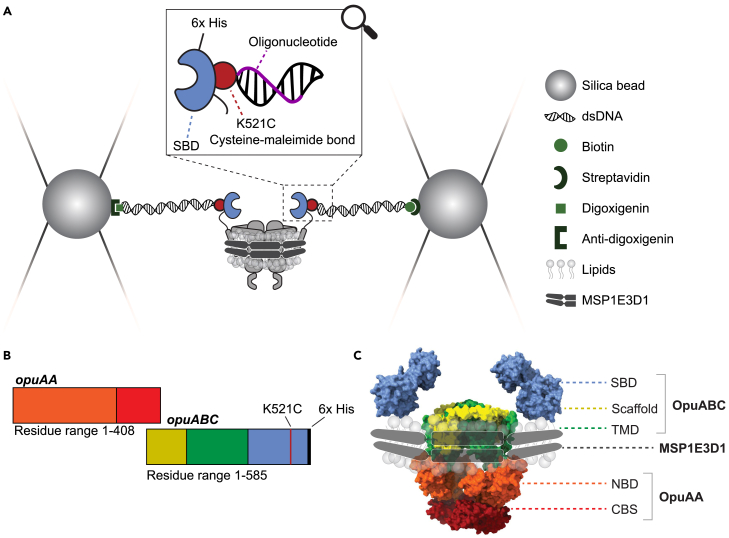
Experimental setup and domain organization of OpuA (A) Schematic of the smOT setup where OpuA is tethered between two beads. (B) Gene organization of OpuA mutant K521C used for labeling. (C) Domain organization of OpuA, which is reconstituted into a lipid nanodisc. The structure of OpuA is based on PDB 7AHH, where the SBDs have been added manually. A and C have been reprinted and adapted from van der Sleen et al.^1^ published under the Creative Commons CC-BY license.

The protocol below describes the specific steps for sample preparation of the membrane protein OpuA reconstituted in lipid nanodiscs for measurements with single-molecule optical tweezers (smOT) (Figure 1A). However, the method is generally applicable for other proteins and membrane-mimicking constructs to measure (un)folding and conformational dynamics with smOT. Using this procedure for purification and reconstitution in lipid nanodiscs, a stable and homogenous sample can be obtained. The protocol additionally includes instructions on how to prepare double-stranded DNA (dsDNA) handles, how to label the protein for smOT measurements making use of cysteine-maleimide chemistry, and how to collect and analyze the smOT data.

For attaching dsDNA handles, we make use of a cysteine mutant of OpuA. OpuA is an oligomer, consisting of two OpuABC and two OpuAA proteins (Figures 1B and 1C).^2^^,^^3^ This means that we only have to introduce one cysteine mutation to create two attachment sites for dsDNA handles, which are used for pulling with smOT. The polypeptide OpuABC contains the substrate-binding domain (SBD), scaffold and the transmembrane domain (TMD). OpuAA consists of the nucleotide-binding domain (NBD) and cystathionine beta-synthase domain (CBS) (Figure 1C). The protein construct has a 6x-His-tag at the C-terminus of OpuABC (Figure 1B) which is used for purification. OpuA does not contain any native cysteines. The mutation K521C has been used as an example here to serve as an attachment site for dsDNA handles, as presented in van der Sleen et al.^1^; however the described procedures are also applicable to mutations in other positions. MSP1E3D1 without His-tag is used as a membrane scaffold protein for nanodisc formation, and was expressed and purified as described previously.^1^

### Large-scale expression

**Timing: 3 days**

The protein of interest is overexpressed in *Lactococcus lactis* Opu401^4^ (derivative of NZ9000 in which the *opuA* genes were deleted) from a pNZ plasmid. The plasmid contains the protein of interest (with the K521C mutation), a nisin inducible promoter and an insert of the chloramphenicol resistance gene.^5^ NZ9000 is one of the most commonly used lactic acid bacterium strains for protein expression. The *opuA* genes were deleted to avoid potential interference of native OpuA when overexpressing from the plasmid. This protocol is based on Sikkema et al.^2^

1. Plate the cells:
  a. Streak the appropriate *L. lactis* strain around 12 p.m. on an agar plate containing M17, 1% (w/v) glucose plus 5 μg/mL chloramphenicol (GM17Cm).
  b. Grow overnight at 30 °C (∼21 h).

***Note:*** M17 is a buffered and enriched medium designed for growth of Lactococcus species. Chloramphenicol is used as an antibiotic and is specific for the plasmid that we used for expression.

2. Prepare the pre-cultures for large scale expression:
  a. At the start of the morning, around 9 a.m., inoculate 2 mL GM17Cm with a *L. lactis* colony. Grow during the day without shaking at 30 °C.
  b. At the end of the afternoon, around 5 p.m., inoculate 50 mL GM17Cm with 5 μL of the culture prepared in the morning.
  c. Grow overnight (∼16 h) without shaking at 30 °C.

***Note:*** On this day, you can prepare and autoclave media, calibrate the pH meter, and autoclave the bioreactor for the next day. The 50 mL overnight culture should not have grown completely dense (OD < 3) the following morning.

3. Grow the cells in the bioreactor:
  a. Transfer the 50 mL overnight culture to the bioreactor containing 2% (w/v) Gistex, 1% (w/v) glucose, 65 mM NaPi pH 7.0 plus 5 μg/mL chloramphenicol.
  b. Grow the cells semi-anaerobically at 30 °C, while stirring at 200 rpm until OD_600_ = 2.0 (∼3 h). Keep the pH ≥ 6.5 using 4 M KOH.

***Note:*** The pH of the medium should start around 7.0. At OD_600_ ∼ 1.0, addition of KOH is required to keep the pH at 6.5.

***Note:*** We switch to Gistex instead of M17 for large scale expression in the bioreactor since this is a cheaper alternative of an enriched medium. The components of Gistex are however not fully defined. Semi-anaerobically is defined as a condition where limited oxygen is available, but is not completely devoid. This means that we grow the cells in a closed bioreactor (or flask, see alternative below) with some air available, however we limit the stirring/shaking speed to prevent excessive aeration, but still prevent sedimentation of cells.

***Alternatives:*** This protocol can for instance be scaled up from a 2 L to a 10 L bioreactor. Inoculate overnight (∼16 h) 250 mL GM17Cm with 25 μL instead and scale up the bioreactor medium accordingly.

***Alternatives:*** It is also possible to grow *L. lactis* on a large scale in flasks instead of a bioreactor. Cells will have to be induced at an OD_600_ of 0.5 instead, due to acidification of the medium by the production of lactic acid by *L. lactis.* Flasks will need to be shaken/stirred slowly to avoid sedimentation of cells (∼80 rpm)*.* However, this should not be too fast since the cells need to be grown semi-anaerobically. The final protein yield per liter of culture will be lower in flasks than when cells are grown in a bioreactor with pH control.

4. Induce with nisin to express protein:
  a. Add 0.0005% (v/v, equals 1:2000 or 1 mL per 2 L of culture) culture supernatant of the nisin A-producing strain (NZ9700) to the culture.^6^
    ***Note:*** This percentage of nisin has been chosen based on expression tests where the yield of the protein is the highest and without degradation (data not shown).
  b. Supplement the medium with additional 1% (w/v) glucose.
  c. Allow the cells to grow for 2 h while keeping the temperature at 30 °C, stirring at 200 rpm, and the pH equal to 6.5.
  d. Place the cells on ice to prevent further growth.
5. Collect the cell pellet:
  a. Centrifuge the entire culture at 6000 *g* (*e.g.*, with a Beckman JLA-9.1000 rotor) for 15 min at 4 °C to pellet the cells.
  b. Remove the supernatant.
  c. Wash the cells by resuspending the pellet in ∼700 mL ice-cold 100 mM KPi, pH 7.5.
    ***Note:*** The volume of the wash buffer can be reduced to ∼10% of the original volume of the cell culture. Keep in mind that centrifugation buckets with a cap require a minimum volume to avoid imploding.
  d. Centrifuge at 6000 *g* (*e.g.*, with a Beckman JLA-9.1000 rotor) for 15 min at 4 °C.
  e. Resuspend the pellet in 50 mM KPi, pH 7.5, supplemented with 20% (v/v) glycerol.
    **CRITICAL:** Make sure the final OD_600_ is 100 – 200.
    **Pause Point:** Cells can be flash frozen in liquid nitrogen and stored at −80 °C for several months. We generally store them in 50 mL tubes filled with maximally 40 mL of cells per tube.

### Crude membrane vesicle preparation

**Timing: 8 h**

Here, we describe the preparation of crude membrane vesicles from cells that expressed our protein of interest. The protocol is based on van den Noort et al.^7^ We first disrupt the cells, then remove the soluble fraction, and wash and collect the crude membrane vesicles.

**CRITICAL:** 20% (v/v) glycerol is been added in each step to prevent dissociation of the OpuAA subunits from the OpuA complex. See^7^ for a more elaborate overview on the conditions to prevent loss of the OpuAA subunits. All steps are performed on ice or in a cooled environment (4°C – 8 °C) to ensure stability of the protein, unless otherwise indicated.

6. Cells and buffer preparation:
  a. If the cells have been stored at −80 °C, thaw them in a beaker of lukewarm water (∼ 40 °C).
    i. Stir or shake the cells gently every minute to make sure that the cells on the periphery will not get too warm while the cells on the inside are still frozen.
      ***Note:*** Thawing takes approximately 15 min.
    ii. Place the cells on ice once they are thawed.
  b. Prepare 200 – 400 mL of 50 mM KPi, pH 7.5, 20% (v/v) glycerol, 1 mM DTT (buffer A).

***Note:*** DTT should be freshly added.

7. Cell disruption:
  a. Add 100 μg/mL DNase, 1 mM DTT and 2 mM MgSO_4_ to the cell suspension.
  b. Slowly add 1 mM PMSF (prepared as a 100 mM stock in isopropanol), while stirring to make sure the PMSF is distributed evenly.
  c. Rupture the cells by passing them twice through a cooled high-pressure homogenizer HPL6 (Maximator GmbH; 30 kPsi) or an equivalent device.
    ***Note:*** Make sure that the Maximator has been washed with buffer A prior to the addition of the cells. Additionally, it is possible to wash the Maximator with some buffer A after breaking the cells to increase the yield.
  d. Add 5 mM Na-EDTA, pH 8.0 to the lysed cells.
  e. Pellet the cell debris by low-speed centrifugation at 15,000 g (*e.g.*, with a Beckman JA-25.50 rotor) for 20 min at 4 °C.
  f. Transfer the supernatant to ultracentrifugation tubes by pouring.
    ***Note:*** Make sure not to take any of the white pellet. Sometimes, a lot of the white pellet comes along when pouring out the supernatant. If this happens, the low-speed centrifugation (Step 7E) can be repeated with the supernatant. If the same is observed more frequently, more DNAse can be added in Step 7A.
8. Isolation of the membrane fraction:
  a. Pellet the membrane fraction by ultracentrifugation at 185,000 g (*e.g.*, with a Beckman 50.2-Ti rotor) for 75 min at 4 °C.
    ***Alternatives:*** The membrane fraction can also be collected by ultracentrifugation at 138,000 g (*e.g.*, with a Beckman 45-Ti rotor) for 135 min at 4 °C.
  b. Remove the supernatant, containing the soluble protein fraction.
  c. Retrieve the pellet.
    i. Add a few mL of buffer A to the centrifugation tube and carefully mix with a pestle to release the pellet.
    ii. Transfer to a potter tube and repeat for all tubes.
    iii. Wash the centrifuge tube a few times with buffer A to retrieve all the material.
  d. Homogenize the membrane fraction.
    i. Add 25 – 50% of the original volume of buffer A.
    ii. Rotate and move the pestle up and down in the potter tube.
    iii. Repeat this until it forms a homogeneous and uniform suspension, and no particles of the membrane pellet are visible anymore.
      **CRITICAL:** Make sure to do this as gently as possible (*i.e.*, try to prevent formation of air bubbles) as this may induce dissociation of OpuAA from the complex.
  e. Transfer the solution to ultracentrifugation tubes and repeat the ultracentrifugation step.
  f. Remove the supernatant, transfer the pellet to a potter tube and homogenize in buffer A. Use 10 mL buffer A per ∼100 mL cells of an OD_600_ of 200.
9. Total protein concentration determination:
  a. Determine the total protein concentration with the Pierce BCA Protein Assay Kit (Thermo Fisher Scientific Inc., Waltham, Massachusetts), using BSA as a standard (0 – 80 μg/mL).
    ***Note:*** Make sure to dilute the protein to an appropriate measurement range. The expected concentration is 8 – 16 mg/mL.
  b. Aliquot the crude membrane vesicles (CMVs) in samples of 18 mg.
    **Pause Point:** The CMVs can be flash frozen in liquid nitrogen and stored at −80 °C for several months when the protein is required for labeling. We have observed that the cysteines are less reactive to maleimides when the CMVs have been stored at −80 °C for more than a year. CMVs that are not used for labeling can be stored for a longer time.

### Synthetic lipid preparation

**Timing: 7 h**

This section describes the preparation liposomes of a synthetic lipid mixture, which can later be used for reconstitution. The protocol is based on Geertsma et al.^8^ In this protocol, we use a lipid composition of 50 mol% 1,2-dioleoyl-*sn*-glycero-3-phosphoethanolamine (DOPE), 12 mol% 1,2-dioleoyl-*sn*-glycero-3-phosphocholine (DOPC), and 38 mol% 1,2-dioleoyl-*sn*-glycero-3-phospho-(1′-rac-glycerol) (DOPG). However, this procedure is also generally applicable for different synthetic lipid mixtures.

**CRITICAL:** Handle all solutions with organic solvent with glass pipettes and use them in a fume hood.

10. Lipid stock preparation in chloroform:
  a. Weigh out the appropriate amount of powdered solid lipid stocks in a glass container for a 25 mg/mL lipid stock. *E.g.*, to prepare 10 aliquots of 250 μL with 25 mg/mL of a 50:12:38 DOPE:DOPC:DOPG lipid mixture, weigh 12.1 mg DOPE, 3.1 mg DOPC and 9.8 mg DOPG.
    ***Alternatives:*** It is possible to prepare more of the 25 mg/mL lipid stock in chloroform than immediately needed. The lipids dissolved in chloroform can be stored in a closed glass container at −20 °C for one year. Add some Teflon tape around the screw lid to avoid evaporation of the chloroform.
    ***Note:*** Make sure to flush the powdered solid lipid stock with liquid nitrogen and store the container using some Teflon tape around the screw lid to avoid oxidation.
  b. Take the appropriate volume of chloroform for a 25 mg/mL stock solution and dissolve the lipids. *E.g.*, to prepare 10 aliquots of 250 μL with 25 mg/mL of a 50:12:38 DOPE:DOPC:DOPG lipid mixture, use the following amounts of chloroform: 0.484 mL DOPE, 0.123 mL DOPC and 0.394 mL DOPG.
  c. Transfer the lipids and mix the solution in a spherical glass flask that is compatible with a rotary evaporator.
  d. Attach the spherical glass flask to the rotary evaporator using a clamp.
  e. Evaporate the chloroform at a pressure of ∼450 mbar at 40 °C while rotating the spherical glass flask.
    **CRITICAL:** The rotation of the flask should be as fast as possible, but without the liquid ‘swinging’. Add some aluminum foil to the outlet to prevent early condensation.
    ***Note:*** The dried lipids should form a semi-opaque uneven layer. The evaporation of chloroform usually takes 30 min to 1 h.
  f. Once the lipids are dried, close the valve to reach maximum vacuum and incubate for 30 min to remove residual chloroform.
11. Preparation of lipids for hydration by using diethyl ether to form an even lipid film:
  a. Dissolve the dried lipids in diethyl ether using the same volume as the original lipid mix solution was prepared with. This can be achieved by adding the diethyl ether to the same flask and rotating the flask on the rotary evaporator without applying vacuum. *E.g.*, when following the amounts stated in Step 10, 1 mL of diethyl ether needs to be added.
  b. Once completely dissolved, close the valve to reach a pressure of ∼ 850 – 900 mbar at 40 °C and allow the solution to dry completely.
    ***Note:*** The evaporation of diethyl ether usually takes ∼ 5 min.
  c. Once dry, close the valve to reach maximum vacuum and incubate for 30 min to remove residual diethyl ether.
    ***Note:*** The lipids should now form a homogeneous film.
  d. To ensure that the diethyl ether is completely removed, detach the flask from the rotary evaporator and gently flow nitrogen gas into the glass flask.
12. Hydration of the lipid film by resuspension in buffer, sizing of the lipid suspension by sonication and storage:
  a. Repeat the following twice to transfer the lipids in buffer to a 15 mL Falcon tube:
    i. 1.25 mL of 50 mM KPi, pH 7.0 to the lipids (this is 50% of the total volume of chloroform in Step 10B).
    ii. Resuspend the lipids by rotating the flask on the rotary evaporator without vacuum.
    iii. Transfer the lipid mixture to a 15 mL Falcon tube.
      ***Note:*** The lipids will not resuspend completely, they will remain as clumps in suspension. To facilitate transfer by pipetting, the lipid mixture in the round bottom flask can be sonicated in a sonication bath to obtain a white suspension.
  b. Place the tube containing the lipids in an ice-water bath and sonicate the lipids to form small unilamellar vesicles (SUVs) using 16 cycles: 15 s on, 45 s off, amplitude 77 μm.
    ***Note:*** After sonication, the solution should be homogeneous without clumps and less opaque than before (however, the extent of clearing may depend on the lipid composition).
  c. Prepare aliquots of 250 μL of the lipid solution in 1.5 mL Eppendorf tubes. Pierce the lid with a syringe needle to release pressure from storage in liquid nitrogen.
  d. Fuse the SUVs to form large multilamellar vesicles (LMVs by freeze-thaw cycles.
    i. Flash freeze the SUVs using liquid nitrogen.
    ii. Allow the samples to thaw at room temperature (∼ 20 °C, ∼ 30 min).
    iii. Repeat this freeze-thaw cycle two more times.
      **Pause Point:** The lipids can be flash frozen in liquid nitrogen and stored at −80 °C or in liquid nitrogen for at least 1 year.

### Detergent preparation

**Timing: 1 h**

*n*-Dodecyl-β-d-maltoside (DDM) is ordered from Glycon Biochemicals GmbH. A stock of 10% (w/v) is prepared by dissolving DDM in Milli-Q water (MQ). Aliquots of 1 mL are prepared to minimize freeze-thawing of the DDM solution to a maximum of two times; the solutions can be stored at −20 °C for several months.

### Maleimide-oligonucleotide preparation

**Timing: 30 min**

Maleimide-oligonucleotides were ordered commercially (Biomers, DE). Upon arrival, the maleimide-oligonucleotides were diluted to 100 pmol/μL in 10 mM Tris-HCl, pH 8.5, 0.1 mM EDTA. 10 μL aliquots were prepared (each aliquot contains 1 nmol of maleimide-oligonucleotides). Aliquots can be stored at −20 °C for several months.

### dsDNA handle preparation

**Timing: 4 h**

dsDNA handles with a contour length of 185 nm were produced in-house utilizing λ-phage DNA bases 10557 – 11100 (544 base pairs) as a template by PCR using Taq polymerase. Primers are listed in the key resources table.^9^^,^^10^

13. Mix the components for the PCR on ice as described in Table 1:

**Table 1:** PCR reaction master mix

Component	Final concentration	Volume
MQ	–	226 μL
5x Taq standard reaction buffer	1x	60 μL
dNTPs (10 mM)	200 μM	6 μL
For primer (100 μM)	1 μM	3 μL
Rev primer (100 μM)	1 μM	3 μL
Template DNA (500 μg/ml)	0.83 μg/μL	0.5 μL
Taq polymerase (5000 units/mL)	25 units/mL	1.5 μL

***Note:*** To produce the dsDNA handles with a biotin modification at the 5′-end, the multi bio primer and linker primer for DNA handles were used in the PCR reaction.

***Note:*** A combination of the multi dig primer and linker primer for DNA handles were used to obtain the digoxigenin modification.

14. Divide the components over the PCR tubes (50 μL per tube, 6 tubes for each dsDNA handle) and start the PCR using the protocol described in Table 2:

**Table 2:** PCR cycling conditions

Steps	Temperature	Time	Cycles
Initial denaturation	95°C	2 min	1 cycle
Denaturation	95°C	15 sec	30 cycles
Annealing	55°C	15 sec	
Elongation	72°C	45 sec	
Final elongation	72°C	5 min	1 cycle
Hold	4°C	infinite
15. Clean the produced dsDNA handles with a PCR clean-up kit (*e.g.*, the NucleoSpin Gel & PCR Clean-up kit from Macherey-Nagel but also other kits can be used).
  a. Follow the instructions (except the elution buffer) provided by the PCR clean-up kit and combine all 6 samples on one column.
  b. Elute with 30 – 40 μL of MQ (rather than elution buffer) after 5 min incubation.
16. Check whether the product has the correct length on an agarose gel (band expected at 544 base pairs).
17. Determine the concentration by Nanodrop.

***Note:*** Expect a concentration around 200 ng/μL.

18. Mix the biotin- and digoxigenin functionalized dsDNA handles 1:1.

**Pause Point:** dsDNA can be stored at −20 °C for several months. Aliquots of slightly more than 200 ng are recommended.

***Alternatives:*** Longer dsDNA handles can also be used. The preparation of these would require different primers. The advantages of longer dsDNA handles include the increased distance of the protein from the laser, reduced potential radiation damage, and increased ease of catching individual protein-DNA constructs. The disadvantage of longer dsDNA handles is the reduced resolution in the force measurements.

### Oxygen scavenging system preparation

**Timing: 3 h**

An oxygen scavenging system for smOT measurements is recommended to prevent damage of the sample due to the production of oxygen free radicals induced by the laser illumination. Generally, we use ∼ 20% laser power (equivalent to ∼ 2 W) of the 10 W laser (1064 nm) on the C-Trap Dymo.

***Note:*** due to power losses in the optical path, the laser power in the sample plane is much lower than the initial laser power indicated above. With a trap 1 split of 50% and 10% laser power, 170 mW in each trap in the sample plane has been measured by the manufacturer of the C-Trap Dymo. At ∼ 20% laser power, therefore ∼ 340 mW is expected in each trap.

We make use of the glucose oxidase/catalase/glucose system (also referred to as GODCAT), which is one of the most commonly used oxygen scavenging systems.^11^ Aliquots of a concentrated stock can be prepared in advance. Shortly before the measurement, 1700 U/mL catalase, 26 U/mL glucose oxidase, and 0.66% (w/v) glucose can be mixed into the measurement buffer.

19. Preparation of 85,000 U/mL catalase:
  ***Note:*** This step describes the preparation of catalase from a suspension of crystals, since the preparation of aliquots worked easier in our hands compared to the powder form.
  a. Transfer the catalase crystals:
    i. Gently mix the suspension of catalase crystals by agitating the bottle.
    ii. Transfer 1,000,000 U of catalase to a 1.5 mL Eppendorf tube (this usually corresponds to ∼ 100 μL of the suspension of catalase crystals).
  b. Wash the catalase crystals:
    ***Note:*** The washing of the crystals is done to remove the thymol preservative in which the crystals are supplied.
    i. Resuspend the catalase in 1 mL of MQ (catalase will not dissolve in MQ).
    ii. Pellet the catalase by centrifugation at 17,000 g for 5 min.
    iii. Discard the supernatant.
    iv. Repeat the above steps four more times.
  c. Finally resuspend the pellet in 1.25 mL of 50 mM KPi, pH 7.0, corresponding to a final concentration of 85,000 U/mL. Dissolving the pellet will take tens of minutes at 37 °C while gently agitating.
  d. Prepare 15 μL aliquots and store at 4 °C for a maximum of 3 months.
20. Preparation of 1300 U/mL glucose oxidase:
  a. Weigh 5.26 mg of glucose oxidase.
  b. Dissolve in 20 mM HEPES-K, pH 7.0, 50 mM KCl, 50% v/v glycerol.
  c. Prepare 15 μL aliquots and store at −20 °C for a maximum of 3 months.
21. Preparation of 33% (w/v) glucose:
  a. Dissolve 330 mg glucose in MQ with a total volume of 1 mL.
  b. Prepare 15 μL aliquots and store at −20 °C for a year.

***Alternatives:*** Alternative oxygen scavengers are: pyranose oxidase/catalase/glucose^12^ or protocatechuic acid (PCA)/protocatechuate-3,4-dioxygenase (PCD).^11^

## Key resources table

**Table:** 

REAGENT or RESOURCE	SOURCE	IDENTIFIER
**Bacterial and virus strains**		

Nisin A-producing strain (NZ9700)	Kuipers et al.^6^	N/A
*L. lactis* Opu401	Biemans-Oldehinkel et al.^4^	N/A

**Chemicals, peptides, and recombinant proteins**		

MSP1E3D1	van der Sleen et al.^1^	N/A
M17 broth	Formedium	M170110
D(+)-glucose anhydrous	Formedium	Glu03
Chloramphenicol	Roth	3886.2
Gistex LS	Strik BV, Eemnes, the Netherlands	N/A
Sodium di-hydrogen phosphate monohydrate (NaH_2_PO_4_·H_2_O)	Sigma-Aldrich	1.06346
Di-sodium hydrogen phosphate (Na_2_HPO_4_)	Sigma-Aldrich	1.06586
Glycerol	Boom	76050772.2500
Liquid nitrogen	N/A	N/A
Dithiothreitol (DTT)	Roth	6908.1
Di-potassium hydrogen phosphate trihydrate (K_2_HPO_4_·3H_2_O)	Sigma-Aldrich	1.05099
Potassium d-hydrogen phosphate (KH_2_PO_4_)	Sigma-Aldrich	1.04873
Deoxyribonuclease I from bovine pancreas (DNase)	Sigma-Aldrich	DN25
Magnesium sulfate (MgSO_4_)	Boom	76051140
Phenylmethylsulfonylfluoride (PMSF)	Roth	6367.2
Ethylenediaminetetraacetic acid disodium salt dihydrate (EDTA-Na_2_·2H_2_O)	Sigma-Aldrich	ED2SS
1,2-dioleoyl-*sn*-glycero-3-phosphoethanolamine (DOPE)	Avanti Polar Lipids, Inc.	850725P
1,2-dioleoyl-*sn*-glycero-3-phosphocholine (DOPC)	Avanti Polar Lipids, Inc.	850375P
1,2-dioleoyl-*sn*-glycero-3-phospho-(1′-rac-glycerol) (DOPG)	Avanti Polar Lipids, Inc.	840475P
Chloroform	Macron	6754–25
Diethyl ether	BioSolve	052805
Nitrogen gas	N/A	N/A
*n*-Dodecyl-β-D-maltoside (DDM)	Glycon Biochemicals GmbH	D97002
Tris	ITW Reagents	A2264
Standard *Taq* reaction buffer	New England Biolabs, US	B9014S
*Taq* DNA polymerase	New England Biolabs, US	M0273
Deoxynucleotide (dNTP) solution mix	New England Biolabs, US	N0447
Potassium chloride (KCl)	Sigma-Aldrich	1.04936
HEPES	Roth	HN77.5
Catalase from bovine liver	Sigma-Aldrich	C30
Glucose oxidase from *Aspergillus niger*	Sigma-Aldrich	G2133
Imidazole	Sigma-Aldrich	56749
Ni^2+^-Sepharose 6 fast flow	Cytiva	17531803
BioBeads SM-2 Adsorbent Media	Bio-Rad	1523920
ROTI GelStain	Roth	3865.1
Agarose SPI	Duchefa Biochemie	A1203
Bovine serum albumin (BSA)	Sigma-Aldrich	A7030
Streptavidin silica particles (⌀ 1–1.4 μm)	Spherotech	SVSIP-10-5
Anti-digoxigenin silica particles (⌀ 1–1.4 μm)	Spherotech	N/A
Mucasol	Schülke & Mayr GmbH	70003439

**Oligonucleotides**		

Multi bio primer: 5′- Biotin-GGCGA(Biotin-dT)CTGG(Biotin-dT)CGTTGATTTG -3′	Metabion	N/A
Multi dig primer: 5′- Dig-GGCGA(Dig-dT)CTGG(Dig-dT)CGTTGATTTG -3′	Metabion	N/A
Linker primer for DNA handles: 5′- CGACTCGCTGGTCTGGTTGAACGTCAGC CCTGCC(dspacer)CCTGCCCGGCTCTGGACAGG -3′	Metabion	N/A
Maleimide-oligonucleotides: 5′-GGCAGGGCTGACGTTCAACCAGACCAGC GAGTCG-Maleimide-3′	Biomers	N/A

**Recombinant DNA**		

λ-phage DNA	New England Biolabs, US	N3013L
pNZOpuAHis_K521C	van den Noort et al.^5^	N/A

**Software and algorithms**		

Igor Pro	https://www.wavemetrics.com	Version 9.0.2

**Other**		

Applikon bioreactor	Getinge	N/A
High-pressure homogenizer HPL6	Maximator GmbH	N/A
Potter-Elvehjem PTFE pestle and glass tube (45 mL)	Sigma-Aldrich	P7984
Potter-Elvehjem PTFE pestle and glass tube (8 mL)	Sigma-Aldrich	P7859
Pierce BCA Protein Assay Kit	Thermo Scientific	23225
Rotary evaporator	BUCHI	N/A
Teflon tape	N/A	N/A
Milli-Q system (ultrapure lab water system)	Merck Millipore	N/A
PCR machine	N/A	N/A
Nanodrop	N/A	N/A
NucleoSpin Gel & PCR Clean-up	Macherey-Nagel	740609
C-Trap Dymo (with short tether piezo tracking)	Lumicks	N/A
NGC chromatography system	Bio-Rad	N/A
Poly-Prep chromatography column (10 mL)	Bio-Rad	7311553
Superdex 200 Increase 10/300 GL	Sigma-Aldrich	GE28-9909-44
High-speed centrifuge: Avanti J-20 XP	Beckman Coulter	N/A
Table-top ultracentrifuge: Optima Max-XP	Beckman Coulter	393315
Ultracentrifuge: Optima XE-90	Beckman Coulter	A94471
JLA-9.1000 fixed-angle aluminum rotor	Beckman Coulter	336754
JA-25.50 fixed-angle rotor	Beckman Coulter	363055
Type 50.2 Ti fixed-angle rotor	Beckman Coulter	337901
Type 45 Ti fixed-angle titanium rotor	Beckman Coulter	339160
MLA-80 fixed-angle rotor package	Beckman Coulter	367096
Ultrasonic batch	Bandlein	N/A
Q125 Probe sonicator	QSonica	5323–15
Probe tip, 5/64″, 200 μL–5mL	QSonica	5323–09
Microscope slide (26 x 76) mm, 1 mm thickness	Knittel Glass	N/A
Cover-slide (22 x 22) mm, 175 μm thickness	Knittel Glass	N/A
Parafilm	N/A	N/A
Heating plate	N/A	N/A

## Step-by-step method details

### Purification and reconstitution of membrane protein OpuA in lipid nanodiscs

**Timing: 6 h + overnight step (∼16 h)**

This section describes the purification of membrane protein OpuA from crude membrane vesicles and reconstitution in lipid nanodiscs to serve as a membrane-mimicking environment. The protocol is based on the optimized purification protocol described by van den Noort et al. (2024).^7^

**CRITICAL:** Avoid redundant agitation and a large dead-volume to minimize the loss of the OpuAA subunit. Also avoid air bubbles when pipetting. All steps are performed on ice or in a cooled environment (4 – 8 °C), unless otherwise indicated.

1. Solubilization of synthetic lipids:
  a. Thaw 250 μL of the 25 mg/mL prepared synthetic lipids at room temperature (∼ 20 °C).
  b. Dilute to 1 mL in 50 mM KPi, pH 7.0 in a 15 mL Falcon tube.
    ***Note:*** The lipid stock is diluted to 1 mL, since this is the minimum volume required for our sonication setup.
  c. Tip-sonicate the lipids in an ice-water bath for 8 cycles: 15 seconds on, 45 seconds off, amplitude 77 μm.
    ***Note:*** Tip-sonication is required since DDM is not able to fully solubilize MLVs within a time period of less than 5 h. If tip-sonication is not used, we have observed a large variation from batch-to-batch preparation in the amounts of lipids available for reconstitution.
  d. Transfer 900 μL of the sonicated lipids to a small glass vial.
  e. Incubate the sonicated lipids at room temperature (∼ 20 °C) in the dark with 100 μL of 10% DDM (w/v).
    ***Note:*** The solubilized lipids can be stored for several hours until the lipids are needed for OpuA reconstitution in lipid nanodiscs.
2. Solubilization of crude membrane vesicles:
  a. Thaw the CMVs.
    **CRITICAL:** This can be done at room temperature (∼ 20 °C), however place them on ice as soon as they are thawed.
  b. Dilute the CMVs on ice in MLA-80 centrifuge tubes to 3 mg/mL in 6 mL. The final mixture should contain 50 mM KPi, pH 7.0–7.5, 20% (v/v) glycerol, 200 mM KCl, 1 mM DTT plus 0.5% (w/v) DDM.
    ***Note:*** An example with CMVs of 18 mg/mL can be found in Table 3. Make sure that the CMVs are added last.
    ***Note:*** Take into account for the calculation that the CMVs are already in 50 mM KPi, pH 7.5, 20% (v/v) glycerol. 3 M KCl is used to compensate for the salt concentration.

**Table 3:** Example of solubilization mixture

Reagent	Final concentration	Amount
MQ	N/A	1,377 μL
50% (v/v) glycerol	20% (v/v)	2,000 μL
4x Ni-buffer (200 mM KPi, pH 7.0, 800 mM KCl)	50 mM KPi, pH 7.0–7.5, 200 mM KCl	1,250 μL
3 M KCl	200 mM	67 μL
1 M DTT	1 mM	6 μL
10% (w/v) DDM	0.5% (w/v)	300 μL
18 mg/mL crude membrane vesicles	3 mg/mL	1,000 μL
**Total**	**N/A**	**6 mL**
  c. Incubate the mixture on ice for 30 min. Mix once by pipetting up and down after 15 min.
  d. Separate the solubilized proteins from the insolubilized fraction by ultracentrifugation at 336,896 g (*e.g.*, with a Beckman MLA-80 rotor) for 20 min at 4 °C.
3. Purification of the protein by Ni^2+^-Sepharose affinity chromatography:
  a. Equilibrate the Ni^2+^-Sepharose resin:
    i. Add 0.5 mL of Ni^2+^-Sepharose (column volume) to a 10 mL disposable chromatography column.
    ii. Wash the resin with 12 column volumes of MQ.
    iii. Equilibrate with 4 column volumes of 50 mM KPi pH 7.0, 200 mM KCl, 20% (v/v) glycerol, 1 mM DTT, 0.04% (w/v) DDM (buffer B) plus 10 mM imidazole.
      ***Note:*** When a fresh batch of Ni^2+^-Sepharose is used, the resin may turn slightly brown upon the addition of DTT. We have not observed any reduced binding of the His-tag to the Ni^2+^-Sepharose in the presence of DTT.
  b. Close the outlet of the column.
  c. Incubate the resin with the solubilized proteins:
    i. Add the supernatant, supplemented with 10 mM imidazole pH 7.5, to the resin and mix by gentle pipetting with a 1 mL pipette.
    ii. Allow the Ni^2+^-Sepharose to sediment in 10 – 20 min.
    iii. Mix the sample a second time and let it sediment again for 10 – 20 min.
      **CRITICAL:** Agitation during incubation with the resin is avoided, because this induces dissociation of OpuAA subunits from the full OpuA complex. Note that avoiding agitation does reduce the total protein yield.
  d. Drain the column and wash twice with 10 column volumes of buffer B plus 50 mM imidazole pH 7.5.
  e. Elute OpuA by adding consecutively one time 0.6 and four times 0.4 column volumes of buffer B plus 200 mM imidazole pH 7.5. Collect the five fractions separately.
    ***Note:*** Most protein is expected in the third and fourth fraction with a concentration around 3 – 5 mg/mL.
4. Nanodisc reconstitution:
  a. Prepare the reconstitution mix in a 1.5 mL Eppendorf tube by mixing 900 μM lipids, 4.5 μM OpuA (tetrameric complex of two times OpuABC and two times OpuAA), 45 μM MSP1E3D1, 1 mM DTT and 50 mM KPi pH 7.0 in a final volume of 700 – 1000 μL.
    ***Note:*** An example with 4 mg/mL OpuA with a 1000 μL reconstitution mixture can be found in Table 4. Make sure that OpuA is added last.
    ***Note:*** Take into account for the calculation of the KPi concentration, that OpuA and the lipids are already in 50 mM KPi, pH 7.0. We use a volume of 700 – 1000 μL in a 1.5 mL Eppendorf tube to minimize the dead-volume in the tube.

**Table 4:** Example of reconstitution mixture

Reagent	Final concentration	Amount
MQ	N/A	361 μL
1 M KPi, pH 7.0	50 mM KPi, pH 7.0	32 μL
7.3 mM (5.63 mg/mL) lipids	900 μM	123 μL
190 μM MSP1E3D1	45 μM	237 μL
1 M DTT	1 mM	1 μL
18.3 μM (4 mg/mL) OpuA	4.5 μM	246 μL
**Total**	**N/A**	**1,000 μL**
  b. Nutate for 1 h at 4 °C.
  c. Add 500 mg semi-dry activated SM-2 BioBeads.
  d. Nutate overnight at 4 °C (∼ 16 h).
    ***Note:*** When weighing the BioBeads, make sure to remove most of the water before weighing.
  e. Remove the BioBeads the following day by transferring the solution with a pipette to a 2 mL Bio-Rad column and collecting the flow through.
  f. Remove the aggregates by centrifugation at 20,817 g for 15 min at 4 °C.
    **CRITICAL:** It is essential to immediately continue labeling the protein on the same day.

### Labeling with maleimide-oligonucleotides

**Timing: 8 h**

This section describes the labeling of the membrane protein in lipid nanodiscs with maleimide-oligonucleotides. These oligonucleotides will later be used as an attachment point for the dsDNA handles to the protein (Figure 1A). The dsDNA handles can later be attached to the beads that can be caught by the laser in the smOT setup, which will be described in the later sections. Additionally, it describes how the sample quality can be assessed. The protocol is based on van den Noort et al.^5^ but has been adapted to apply for maleimide-oligonucleotides (instead of fluorophores).

5. Removal of empty nanodiscs and labeling with maleimide-oligonucleotides:
  a. Equilibrate the Ni^2+^-Sepharose resin:
    i. Add 0.1 mL of Ni^2+^-Sepharose (column volume) to a 2 mL disposable chromatography column.
    ii. Wash the resin two times with 1 mL MQ.
    iii. Was the resin subsequently with 1 mL of 50 mM KPi pH 7.0 supplemented with 1 mM DTT.
  b. Load the reconstituted protein on the column and collect the flow through.
  c. Reapply the flow through two times to the column to bind most of the protein.
  d. Wash the column twice with 0.5 mL buffer C (300 mM KCl, 20 mM HEPES-K pH 7.0).
    ***Note:*** Make sure to rinse the sides; residual DTT can irreversibly react with the maleimide-oligonucleotides and prevents attachment to the cysteines.
  e. During the washing step, dilute the maleimide-oligonucleotides to 100 μL in buffer C. Use 1.1x the amount of maleimide-oligonucleotides to the amount of cysteines.
    ***Note:*** Make sure to add the buffer dropwise, to make sure that there is no precipitation.
  f. Close the column. Add the diluted maleimide-oligonucleotides to the column and mix gently with the resin.
    **CRITICAL:** To make sure there is no leakage, additionally wrap the outlets with parafilm. Store the column in aluminum foil on ice (without shaking). Make sure that the column remains straight up. Incubate for 4 h.
  g. Wash the unbound maleimide-oligonucleotides away twice with 1 mL buffer C supplemented with 25 mM imidazole.
    ***Note:*** We have observed that unreacted nucleotides can form multimers and elute on a size-exclusion column in several peaks (Figure 2A). We have found that the largest peak elutes around the elution volume of a globular protein of 50 kDa. The elution volume may vary depending on the buffer used (and the SEC column and system). This step is particularly important if your protein is expected to elute around the same volume.
    **Figure 2:**
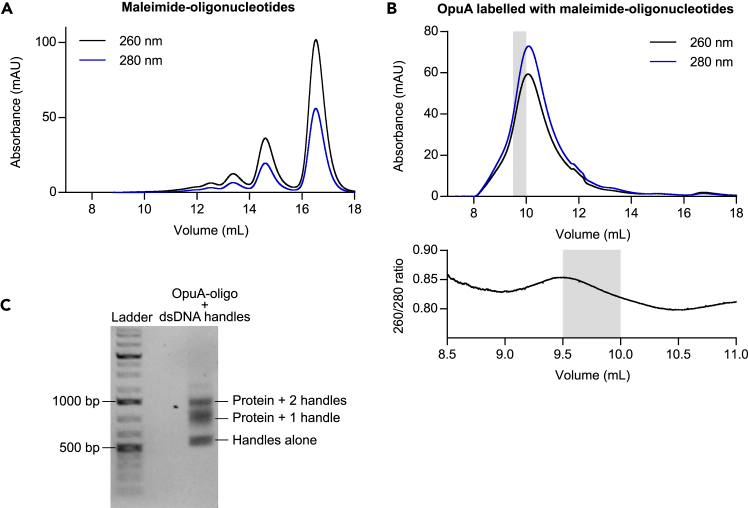
Labeling of OpuA with maleimide-oligonucleotides and dsDNA handles SEC profile of (A) maleimide-oligonucleotides on their own, and (B) OpuA labeled with maleimide-oligonucleotides. The fraction collected for further experiments have been highlighted in gray. (C) Agarose gel after incubation of dsDNA handles with OpuA which has been labeled with maleimide-oligonucleotides.
  h. Elute the protein with 0.4 mL buffer C supplemented with 200 mM imidazole.
    ***Alternatives:*** Instead of DTT, TCEP can be used as a reducing agent. TCEP reversibly interacts with cysteines; therefore TCEP can still be present in the labeling reaction without loss of labeling efficiency.
6. Purification by size-exclusion chromatography (SEC) to separate the protein with either no, one or two maleimide-oligonucleotides attached:
  a. Equilibrate the SEC column (Superdex 200 Increase 10/300 GL) with buffer C.
  b. Load the sample on the column, track where the protein elutes by the absorbance at 260 and 280 nm (Figure 2B). Collect the protein in fractions of 0.5 mL.

***Note:*** The 260/280 ratio can be used to check which elution fraction contains the protein with the maleimide-oligonucleotides attached, since a 260/280 ratio of 0.6 is expected for pure proteins (containing Trp residues) and 260/280 ratio of 1.8 is expected for oligonucleotides. This effect is more pronounced if smaller proteins are labeled. The fraction with the highest 260/280 ratio contains the labeled protein.

***Note:*** If the unreacted maleimide-oligonucleotides are not properly washed away, these contaminants may also show up in the SEC profile. Depending on the size of the protein, the SEC column and buffer, three different protein fractions may be observed when tracking the absorbance: protein without maleimide-oligonucleotides, protein with one maleimide-oligonucleotide attached and protein with two maleimide-oligonucleotides attached.

**Pause Point:** The protein fractions can be stored at −80 °C for several months after flash-freezing in liquid nitrogen. Aliquots of 10 – 20 μL should be sufficient for one day of experiments with the optical tweezers.

***Note:*** Storage conditions may vary from protein to protein. Buffer C was found to be appropriate for our protein after tests with an activity assay; however, some proteins may require 10% (v/v) glycerol for proper storage.

7. Test for labeling by attachment of dsDNA handles to the protein labeled with maleimide-oligonucleotides:
  a. Incubate 100 ng of dsDNA handles with protein from the SEC elution fractions that contain protein with two maleimide-oligonucleotides attached (as collected in Step 6B).
    ***Note:*** To determine the optimal ratio of dsDNA handles to oligonucleotide-protein, it is recommended to test different ratios. For OpuA, 10 μL of ∼0.2 mg/mL labeled-protein incubated O.N. on ice with 100 ng handles works well. We have observed for other proteins, that the incubation time can be decreased to 30 min at RT.
  b. Add loading dye and run on a 1% (w/v) agarose gel with a DNA dye (*e.g.*, ROTI GelStain, ROTH).
    ***Note:*** Each sample will have at least one band (handles alone), possibly two (handles alone, protein + 1 handle), or ideally, three (handles alone, protein + 1 handle, protein + 2 handles) (Figure 2C). The sample that should be chosen is the one which shows three bands with the highest protein + 2 handles:protein + 1 handle ratio.
    ***Note:*** Depending on the protein of interest, the bands may run slightly differently on an agarose gel.

### Sample preparation for smOT

**Timing: 1 h (excluding incubation of labeled protein with dsDNA handles)**

**Figure 3:**
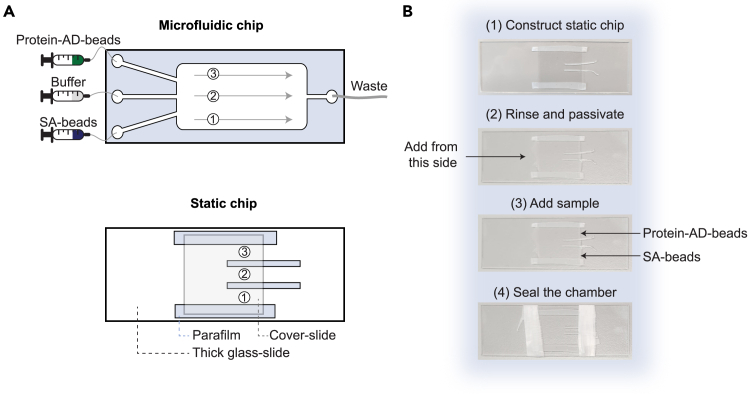
Setup of the microfluidic and static chip (A) Schematic of a microfluidic chip and a static chip. Both chips contain three different channels/chambers containing (1) streptavidin-coated beads, (2) buffer with an oxygen scavenger system, and (3) anti-digoxigenin-coated beads with labeled protein. (B) Step-by-step guide to prepare a static chip.

For smOT measurements using the microfluidic system of Lumicks of the C-Trap Dymo, we make use of three different channels (Figure 3A): channel 1 contains streptavidin-coated beads, channel 2 contains the measurement buffer with the oxygen scavenger system, and channel 3 contains the anti-digoxigenin-coated beads with the labeled protein. This section describes how to prepare the sample shortly before the smOT measurement. The amounts described are sufficient for 1 – 2 h of measurements. An experienced user may reduce the total volumes by 50%, since the volume required depends on the bead-catching efficiency of the user.

8. Removal of oxygen in the buffer:
  a. Filter the buffer with a 0.2 μm filter.
  b. Sonicate the buffer for 10 min.

***Note:*** Approximately 20 mL of buffer should be sufficient for a day of measurement.

9. Preparation of the microfluidic system:
  **CRITICAL:** Make sure there is no pressure on the microfluidic system before removing or adding liquid to the syringes.
  a. Remove the 70% ethanol (or other storing liquid) from the inside of the syringes.
    **CRITICAL:** Do not remove all of the liquid from the syringe – only use the pipette tip to remove the liquid around the edge of the syringe, not from inside the connector – if you do this, you risk introducing air bubbles into the system. To remove an air bubble, pressure waves (series of pressure changes in the applied pressure) may help. Alternatively, the system can be flushed with 70% ethanol.
  b. Add 1 mL of MQ into each syringe.
  c. Run 500 μL through the system at 1.8 bar to remove the ethanol from the chip.
    ***Note:*** Take a close look at the speed at which the liquid-level reduces and check whether there are leaks. At this point, you can identify whether some channels may be clogged. It is required to have equal flow in all channels, since the different liquid streams in the chip are separated by laminar flow.
  d. Stop the flow on the system. Repeat the previous two steps with 1 mL of the measurement buffer.
    ***Optional:*** If required, it is possible to passivate the system with BSA prior to this step. Passivation is done to prevent non-specific binding of proteins to the surface. This can be achieved by flowing 1 mg/mL BSA in MQ through the system at a low flow (0.2 bar) for 20 min.
10. Preparation of solution for channel 1: streptavidin-coated beads (SA-beads)
  a. Vortex the beads for 30 – 60 s to prevent clumps of beads in the sample.
  b. Dilute the SA-beads in the measurement buffer.
    ***Note:*** A good amount to start with is 1 μL of bead stock (1% w/v stock) in a 500 μL buffer.
    **CRITICAL:** Make sure to pipette the beads into the buffer and pipette multiple times up and down to make sure as much beads as possible are transferred.
  c. Vortex the suspension.
11. Preparation of solution for channel 2: measurement buffer with oxygen scavenger system
  **CRITICAL:** Regularly prepare a fresh oxygen scavenger system during a measurement day, since the solution slowly acidifies. It has been shown that the pH may drop more than 0.5 pH units within 1 h in 20 mM Tris-HCl pH 7, 50 mM NaCl.^12^ A good practice is to freshly mix and exchange the oxygen scavenger system every hour.
  a. Mix the components in the measurement buffer according to Table 5.

**Table 5:** Oxygen scavenger system

Reagent	Final concentration	Amount
85,000 U/mL catalase	1,700 U/mL	10.6 μL
1300 U/mL glucose oxidase	26 U/mL	10.6 μL
33% (w/v) glucose	0.66% (w/v)	10.6 μL
Measurement buffer	N/A	498.2 μL
**Total**	**N/A**	**530 μL**
  b. Filter the solution with a 0.2 μm filter.
    ***Note:*** Make sure to use a small filter (*e.g.*, with a diameter of 15 mm), since there is always some dead-volume when filtering.
12. Preparation of solution for channel 3: anti-digoxigenin-coated beads with labeled protein (protein-AD-beads)
  a. Incubate the labeled protein with 200 ng of dsDNA handles.
    ***Note:*** The appropriate incubation time, temperature and ratio have been determined during Step 7. For OpuA, it is important to keep the sample as long as possible in a cooled environment (4°C– 8 °C).
  b. Dilute the incubated sample with buffer, since highly concentrated samples behave poorly in single molecule experiments.
    ***Note:*** For our sample preparation, a 25 times dilution (2 μL of the incubated sample diluted in 48 μL of measurement buffer, equivalent to 8 μg/mL protein) worked well after optimization. This is judged based on the amount of (single) tethers obtained during measurement. We aim to have successful tethering for every three bead pairs tested.
  c. Vortex the AD-beads for 30 – 60 s to prevent clumps of beads in the sample.
  d. Mix 20 μL of the prediluted oligo-protein handle mix with 10 μL beads. Incubate for 10 min at RT.
    ***Note:*** This ratio can also be optimized.
    ***Note:*** Mix the components gently by pipetting up and down and not by vortexing, to ensure the stability of the protein.
  e. Dilute the protein-AD-beads mixture in 500 μL of measurement buffer.
13. Remove the measurement buffer from the syringes of the microfluidic system and add the prepared solutions in the appropriate channels. Catch beads, calibrate, tether the construct between the beads and measure the protein. The procedure is more elaborately described in the next section “smOt measurements”.
14. Cleaning of the microfluidic system after measurements:
  **CRITICAL:** Make sure there is no pressure on the microfluidic system before removing or adding liquid to the syringes.
  a. Remove the solutions from the inside of the syringes as described in Step 9B.
  b. Add 1 mL of MQ into each syringe.
  c. Run 0.5 mL through the system at 1.8 bar to remove the samples from the chip.
  d. Stop the flow on the system.
  e. Repeat the previous two steps with 5% (v/v) mucasol solution.
  f. Apply pressure waves by consecutively applying 1.8 bar and removing the pressure by venting to check whether there are still beads visible. Repeat this step if there are still a lot of residual beads present.
    ***Note:*** Mucasol may also form bubbles which are visible on the bright-field camera; a non-trained user may confuse them with beads.
  g. Repeat Step 14B and 14C with MQ.
  h. Repeat Step 14B, add 2 mL of 70% ethanol, flush 0.5 mL and remove the pressure.
    **CRITICAL:** It is important to always pipette with the same pipette and same size pipette tip, and time every incubation step to obtain reproducible results, since the measurement is very sensitive to small variations in sample preparation.
    ***Note:*** In this protocol, we have used silica beads. Since their density is higher than the aqueous buffer, they tend to sink over time. An alternative is the use of (cheaper) polystyrene beads. Polystyrene beads may however cause more laser-induced damage to the protein due to a production of free radicals.^13^
    ***Alternatives:*** The protocol above describes sample preparation for the microfluidic system of Lumicks. Alternatively, a static chip can be used. The main advantage of a static chip is that less sample is required. A disadvantage is that the chip cannot be reused and should be exchanged every hour due to limited time-use of the oxygen scavenger system and the sample. The static chip can be prepared in the following manner:
15. The static chip consists of one large microscope slide (24 x 50 x 1 mm), two small and two large strips of parafilm, and a small cover-slide (18 x 18 x 0.175 mm). The chamber will have a total volume of approximately 30 – 50 μL (Figure 3A).
  a. Construct the static chip:
    i. Use two slides and two small strips of parafilm to construct the static chip, as shown in Figure 3B.
    ii. Fix the chip by melting the parafilm by placing the chip on a hot plate set at 80 °C.
    iii. Gently press the cover-slide onto the microscope slide with a clean glass slide.
      ***Note:*** The cover-slide is properly attached when the parafilm goes from opaque to transparent.
  b. Rinse the measurement chamber in steps of 120 μL:
    i. Flush the chamber twice with MQ.
    ii. Flush twice filtered 1 mg/mL of BSA.
    iii. Incubate the BSA on the surface for 5 min.
    iv. Flush the chamber with the measurement buffer.
    v. Flush the chamber with the oxygen scavenger system in the measurement buffer.
      **CRITICAL:** When flushing the system, make sure to add buffer from the side with the large opening and not from the individual channels to prevent air bubbles in the chip.
  c. Add 5 μL of each bead solution, as prepared in Step 10 and 12, to the individual channels.
    ***Note:*** If a significant amount of beads are found in the middle channel when checking in the bright field of the optical tweezers instrument, reduce the volume added to the channel.
  d. Seal the measurement chamber:
    i. Add vacuum grease to the two open sides.
    ii. Cover the grease with some parafilm.
      ***Note:*** parafilm is added to prevent potential contamination by the grease onto other surfaces.
      ***Alternatives:*** Instead of parafilm, also double-sided tape can be used. Additionally, instead of the thick microscope slide, a thinner slide can be used (0.175 mm thickness), however this may decrease the mechanical stability of the chip.
      ***Note:*** When working with the static chip in the C-Trap Dymo, using the thick microscope slide (1 mm thickness), make sure the thin cover-slide is on the bottom (near the objective) and the thick microscope slide on top (near the condenser).

### smOT measurements

**Timing: 2 h per measurement session**

This section describes the steps required for the smOT measurements using a system with two optical traps (C-Trap Dymo, Lumicks) and the Lumicks software Bluelake. This protocol has been written in such a way that it may also be applicable for users of other instruments. The protocol includes the catching of beads, calibration, tethering, and the actual protein measurement.

16. Bead catching:
  a. Move to channel 1 (Figure 3A). Catch one streptavidin-coated bead in trap 1.
  b. Move to channel 3. Catch one anti-digoxigenin-coated bead with protein attached in trap 2.
  c. Move with both traps into the channel that contains the oxygen scavenger system in the measurement buffer (channel 2).
17. Calibration of the beads:
  a. Check that the beads are recognized by the video tracking software.
    **CRITICAL:** Since the software recognizes only the middle of the template and not the middle of the beads, make sure that the bead tracking ROI is carefully drawn around the bead.
  b. Move the beads, so they are horizontally aligned with a distance of approximately 5 μm between each other.
    ***Note:*** This distance is required to avoid crosstalk between the two trapping lasers.
  c. Perform a force calibration on the beads to determine the trap stiffness by fitting a power spectrum of the voltage measured by a position sensitive diode.^14^^,^^15^^,^^16^^,^^17^^,^^18^
    **CRITICAL:** Make sure to enter the correct values of the bead diameter prior calibration. This value should be supplied by the manufacturer of the beads. A trap stiffness (κ) of around 0.2–0.3 pN/nm is expected at an overall trapping power of ∼ 20% with a trap 1 split of 50% using the C-Trap Dymo (equivalent to 2 W initial laser power) with silica beads with a diameter of 1 μm. If the trap stiffness is too low, it is recommended to increase the laser power.
  d. Set the force to zero and enable piezo tracking.
    ***Note:*** Piezo tracking is required for our dsDNA handles (of 370 nm total length), since the bead templates will start to overlap when the beads are very close to each other and therefore video tracking (the default for bead tracking in the C-Trap Dymo) will fail. Another advantage of piezo tracking is the higher time resolution (78 kHz for piezo tracking vs 10 – 100 Hz for video tracking with the C-Trap Dymo).
18. Tethering of the construct between the beads:
  a. Move the beads towards each other until the force reaches a maximum (the exact force may be dependent on the buffer and the setup, in our case it is around 10 – 30 pN). It is recommended to do this at a constant velocity of 100 – 500 nm/s.
    ***Note:*** We also refer to this point as the “bead contact point” (Figure 4A). The force is created due to repulsion of the beads to each other and the crosstalk between the two trapping lasers. This approach curve is used to baseline the data, *i.e.*, the forces are subtracted from the traces to make the subsequent data analysis possible.
    **Figure 4:**
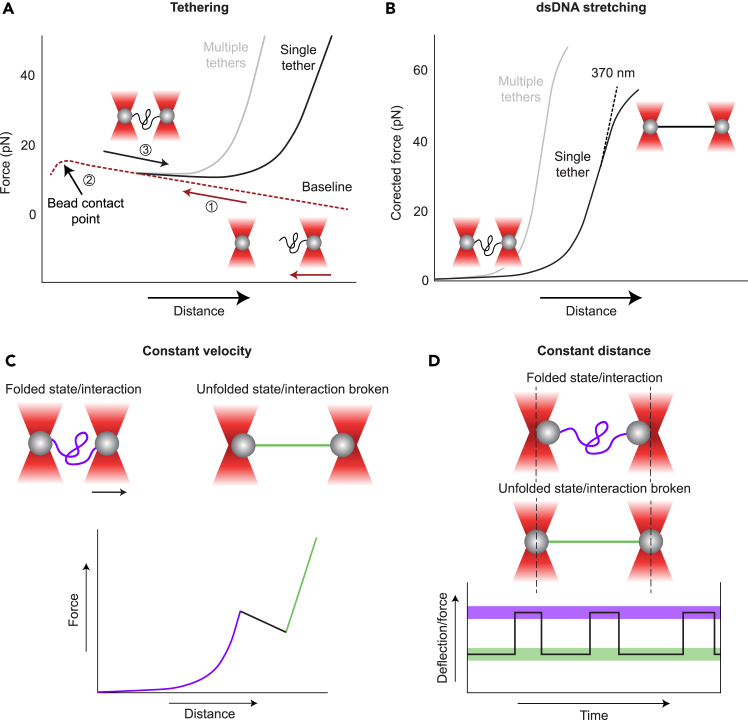
Schematic of smOT measurements (A) Schematic of tethering of a protein construct in between two beads. First, the beads are approached to each other until the bead contact point. Then, the beads are retracted to check whether a tether has been formed. This can be observed as an increase in force relative to the baseline. (B) Stretching of dsDNA with a contour length of 370 nm, fitted with a worm-like chain model after subtraction of the baseline. Multiple dsDNA tethers show a stiffer behavior. (C) Schematic of a constant velocity experiment, where one of the traps is moved at a constant velocity. If a domain unfolds or an interaction is broken, this is observed as a rip in the force-distance plot. (D) Schematic of a constant distance experiment, where both traps are held at the same position and the bead moves within the trap.
  b. Now retract for 100 – 200 nm (NB this length will vary depending on the handle length used) to check for protein-DNA constructs tethered between the beads and repeat until an increase in force is observed. A recommended pulling velocity is 500 nm/s.
    ***Note:*** In Bluelake, an extensible worm-like chain (WLC) fit with a contour length of 370 nm and a persistence length of 20 nm can be plotted as a reference guide. This plot may help to identify whether a single or multiple tethers have been caught, since a more stiff behavior is expected from multiple tethers (Figures 4A and 4B).
    **CRITICAL:** Make sure that you do not pull the beads too far apart when ‘fishing’ for tethers. This may already unfold your protein or break interactions that cannot be recovered. It is recommended to set coordinates for the right pulling distance by using the ‘ping-pong’ feature in Bluelake. Usually, the first bead pairs caught on the measurement day are used to set the correct coordinates. Keep in mind that these may differ slightly from day to day due to some drift, and there can be variation in bead sizes in a given batch (the expected size range is specified by the bead manufacturer).
    ***Note:*** After catching a tether, make sure to move the beads towards each other so there is no force on the tether. This is done to prevent early unfolding of the protein. However, do not move too close since the beads may start sticking to each other and potentially fall into a single trap, making it impossible to use this bead pair again.
19. Protein measurement:
  a. Constant velocity traces:
    ***Note:*** Force-data is often gathered using constant velocity traces (Figure 4C). A good starting point is a velocity of 20 nm/s, where the construct is consecutively stretched and relaxed with a ‘ping-pong’, but the choice of velocity will depend on the protein studied and the research question. This velocity is equivalent to a loading rate of ∼ 5 pN/s. We have found 20 nm/s to provide a good balance between spatio-temporal resolution and being able to obtain a reasonable number of experimental traces. Here, the start- and end-point of the traps are pre-defined, with a 1 s pause at each start- and end-point. The timing of this pause may be adapted depending on the protein and research question. Events, such as domain-domain interactions and protein unfolding, will appear as a rip in the force-distance curve.
    i. Define the start-point of the ping-pong where the tether is fully relaxed (almost no force is exerted on the protein), but the beads are not touching.
      ***Note:*** With our setup, this is where the beads are around 100 nm apart from each other, relative to the bead contact point (this point was defined when tethering).
      ***Note:*** There may be some residual force on the beads measured when the tether is fully relaxed due to the interaction of the beads. To determine whether there is no force on the tether, subtract the baseline (which is determined in the approach curve).
    ii. Measure the construct: Set the end-point of this ping-pong to a short distance (*e.g.*, 300 nm from the start-point). Gather 5 of the same curves at 20 nm/s. Increase the distance by 50 nm and gather 5 of the same curves at 20 nm/s. Repeat the above steps until the tether is disrupted.
      ***Note:*** When measuring a new construct, start with a short distance. When knowing at which distance interesting events happen, it is possible to start at longer distances.
      ***Note:*** The pulling velocity influences the observable unfolding and refolding behaviors of the protein. Unfolding occurs at lower forces when pulled at a lower velocity.^19^^,^^20^ Additionally, the refolding velocity and the time the tether spends in the relaxed state after unfolding may alter the refolding properties of the protein.^21^
  b. Constant distance or constant force traces:
    ***Note:*** Constant distance or constant force measurements can be performed by keeping the traps at a constant position or applying a constant force feedback loop (Figure 4D). This type of data is typically only recorded after defining at which trap distances certain transitions take place based on constant velocity data. Data of a transition is recorded within a set range of trap distances (or forces). The advantage of constant distance and constant force measurements is the added information on kinetic properties, *e.g.*, the (un)folding rate at zero force and the distance to the transition state.^22^^,^^23^ Depending on the protein, also a better resolution of states at low force and a better resolution of length changes can be achieved with this measurement method. Below we present an example protocol for collecting constant distance data over a range of distances around a transition of interest (*e.g.*, the unfolding of a specific protein domain).
    i. Set the trap distance at the lowest distance the transition of interest has been observed. Record for 30 sec.
      ***Note:*** A transition can be observed as a change in the deflection, *i.e.*, a change in the force measured, at the same distance between the traps.
    ii. Increase the trap distance by 10 nm and record for 30 s. Repeat until the transition is not observed anymore.
      ***Note:*** The recording time and increase of the trap distance are dependent on the properties of the transition of interest. Recording time for fast transitions can be shorter than for slow transitions. The increase of trap distance may be smaller/larger depending on the range of forces the transition occurs.
  c. Export the recorded data.

### Data analysis

**Timing: 1–2 days depending on experiment**

This section describes some of the options for the analysis of the force data (constant velocity and constant distance traces). In short, first the baseline is subtracted, then the dsDNA stretching is fitted, and lastly, rips in the data are analyzed where information on the force and distances are extracted. More details on the used models can be found in Jahn et al.^24^ From this data, among many other information, for instance (un)folding pathways can be revealed, misfolding and effects of substrates/buffers on the protein can be studied and domain-domain interactions can be revealed. See Corrêa et al.^23^ for a more elaborate overview.

20. Preparation of data:
  a. Load the .hdf5 file in the analysis software (*e.g.*, Igor Pro).
  b. Subtract the force measured in the approach-curve (baseline) as recorded in Step 18A from all obtained force-distance curves.

***Note:*** This approach-curve holds information on the forces excreted on the beads without a tether and includes the force induced by the repulsion of the two beads and the interference of the two traps due to overlap.

21. Analysis of constant velocity traces:
  a. Fit the stretching of the dsDNA handles with an extensible WLC using the following parameters: a contour length of 370 nm, persistence length of 20 nm, temperature of 297 K and a Hookean contribution (K-value) of 400 – 1200 pN.
    ***Note:*** These parameters have been chosen based on an earlier publication for the same dsDNA handles used with a different protein sample.^24^ The K-value depends on the buffer conditions, since ions can influence the mechanical properties of DNA.
    ***Note:*** At this stage, it is possible to determine whether one or multiple tethers have been measured. In case of multiple tethers, the stiffness is much higher resulting in a much steeper increase of the force as function of the distance. Additionally, the contour length is smaller. The latter results in a force-distance curve where there is already a higher force exerted on the tether at a shorter distance (Figure 4B).
  b. Identify rips in the force-distance curves and fit with a standard WLC fit with a persistence length of 0.7 nm.
    ***Note:*** Rips in the force-distance curves are caused by breaking of domain-domain interactions and/or unfolding of the protein (Figures 5A and 5B). This results in a gain of the contour length. The distance changes related to this are fitted with a standard WLC fit, where a persistence length of 0.7 nm of the unfolded protein is used.
    ***Note:*** Tether breakages (*e.g.*, when the anti-digoxigenin interaction with digoxigenin is broken) will appear different then the rips described above. If the tether breaks, no more force will be exerted on the construct when pulling the beads further apart, whereas with the described rips, the force will later continue to rise.

**Figure 5:**
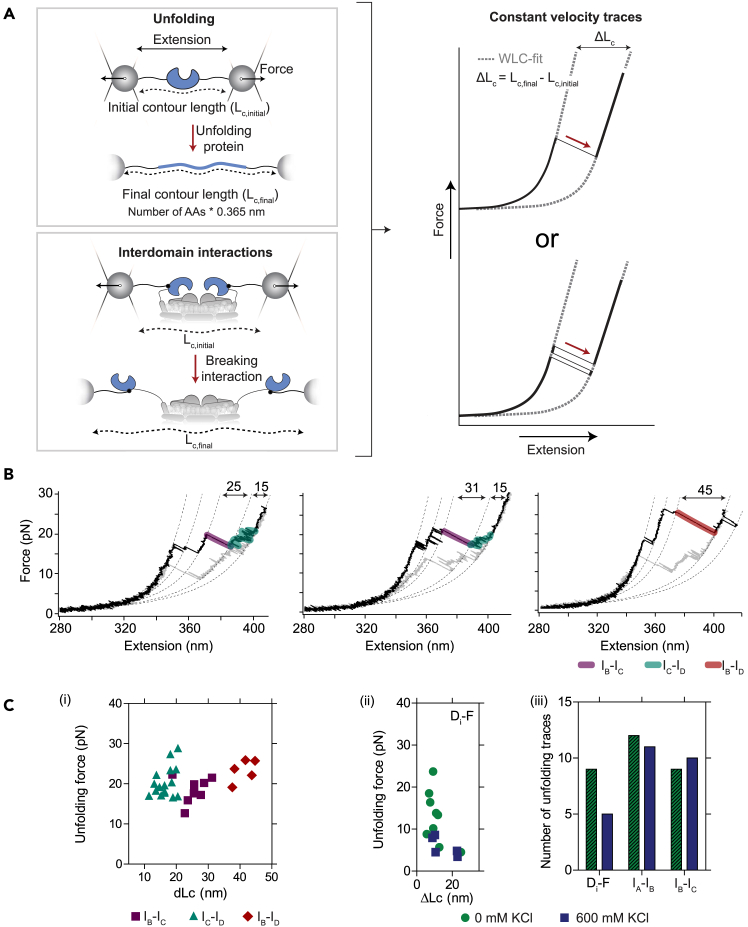
Data analysis of constant velocity measurements (A) Schematic of events that result in contour length changes. An event can be identified by comparing the measured contour length change (after fitting with a worm-like chain model) and the predicted contour length changes by subtracting the final and initial contour length. An event can occur immediately (top-right) or after some fluctuations between the folded and unfolded state, or the associated and dissociated state (bottom-right). The worm-like chain fitting in both cases should result in the same measured change in contour length. Figure has been reprinted and adapted from van der Sleen et al.^1^ published under the Creative Commons CC-BY license. (B) Force-distance curves from constant velocity measurements, data from van der Sleen et al.^1^ of OpuA in 50 mM HEPES-K pH 7.0, 600 mM KCl. Unfolding traces are shown in black and folding traces in gray. Event I_B_-I_C_ (purple) occurs immediately, whereas event I_C_-I_D_ (teal) occurs after some fluctuations, as presented in Figure 5A. Both events may also appear as a single rip (event I_B_-I_D_, red). WLC fits are shown with dotted lines and the change in contour length (ΔLc) is reported in nm. (C) Example results after data analysis of constant velocity traces. (i) Unfolding of the same structural element may appear as a single event or in multiple steps. The unfolding force (ii) or frequency (iii) of an unfolding or dissociation event may change under different conditions. Data presented here has been replotted from van der Sleen et al.^1^ recorded in 50 mM HEPES-K pH 7.0 with 0 or 600 mM KCl.
  c. Identify to which event the contour length gain corresponds.
    ***Note:*** The predicted length changes for each event can be determined based on a structure (Figure 5A). For protein unfolding, the expected contour length change of an unfolding domain can be calculated with the following formula:
    $$\Delta L_{c}=L_{c,final}-L_{c,initial}$$
    For unfolding of a protein domain, the following holds:
    $$L_{c,final}=\#AAs*0.365$$
    where *L*_*c,final*_ is the final contour length (in nm), *#AAs* is the number of amino acids in the domain, *0.365* is the average length of the backbone of a single amino acid (in nm), and *L*_*c,initial*_ is the initial distance (in nm) between the N- and C-termini of the domain from the structure. Assignment is done based on the expected length changes and the expected order of disruption/unfolding based on the stability of the interaction, on average. However, this assignment may not always be trivial (see also Problem 11).
    ***Note:*** The pulling geometry may affect the unfolding force. It may also be possible to separate events based on their reversibility (refolding or re-association rate).
  d. Analyze the obtained data.
    ***Note:*** There are several pieces of information that can be learned from the constant velocity traces, of which some example traces are shown in Figure 5B. We have visualized some of the most commonly used analysis in Figure 5C. This includes: (i) determining whether unfolding of a domain occurs in a single rip or multiple rips, (ii) observing changes in the unfolding force of an event due to the addition of a compound or changing the buffer, and (iii) comparison of the frequency of an event under different conditions. In example (i), the contour length change of the single step is the same as the sum of the contour length changes of the multiple rips. Data shown in Figure 5C is a result from analyzing force-distance curves of which a selection is shown in Figure 5B. By comparing constant velocity traces in low and high salt buffers, it has for instance been shown that the SBDs of OpuA interact in a salt dependent manner after assigning events (Figure 5Cii).^1^ This may be related to the salt dependent regulation of activity of this protein.
22. Analysis of constant distance traces to derive kinetic properties:
  a. Make sure to have fitted a constant velocity trace in the same experiment to be able to assign states correctly before analyzing the constant distance measurements.
  b. Identify the transitions in each constant distance measurement by Hidden Markov Modelling.^25^
  c. Assign the transitions to the events identified from the constant velocity data.
    i. Compare the force at which the transition takes place with the constant velocity data.
    ii. Compare the contour length change of the transition with the contour length changes calculated from the WLC model for constant velocity unfolding traces of the same molecule.
  d. Derive kinetic properties from the data: the energy difference between states and the transition rate between states at zero applied force.
    ***Note:*** To achieve this, multiple measurement points need to be taken at different forces where the transition is visible. From the example traces shown in Figure 6A, the energy difference and rates have been obtained (Figure 6B). For more details, see.^24^
    i. Derive the energy difference (Figure 6Bii): Obtain the probability of the protein being in a specific state from summing the dwell times in this state and dividing it by the total trace time. Then, derive the energy difference between the two states, based on equilibrium statistical mechanics.^24^
    ii. Obtain the rates at zero applied force: Obtain unfolding and refolding rates (Figure 6Biii) from the Hidden Markov analysis or by fitting dwell time histograms with single exponentials. Then, fit the determined rates at different forces an extension of the Zhurkov-Bell-Evans-Richie model to obtain the rates at zero applied force.^20^^,^^26^

**Figure 6:**
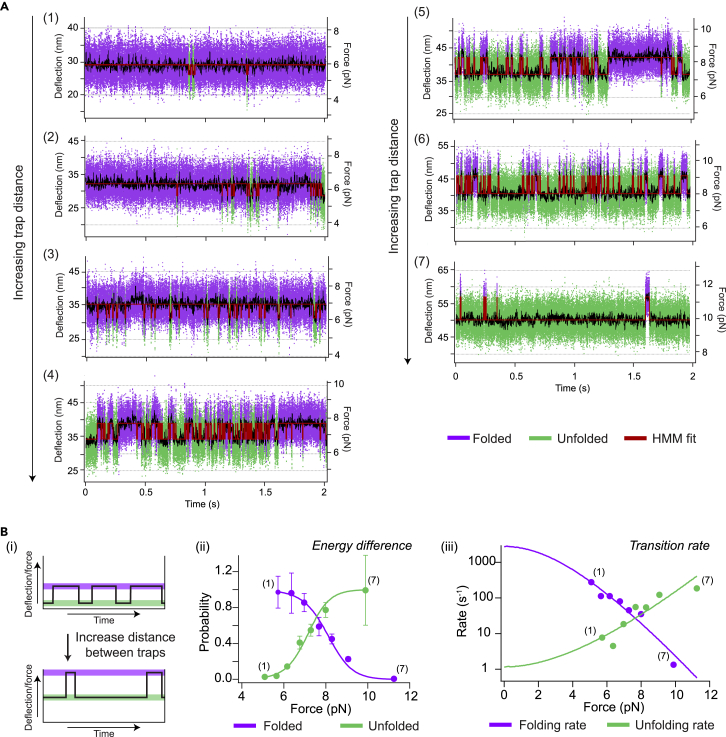
Data analysis of constant distance measurements (A) Constant distance traces belonging to panel (B). Numbering is according to the measurement order in which the trap distance is increased between every panel. The traces have been cropped to show 2 s of the measurement for each trap distance. Increasing the trap distance shifts the equilibrium from a folded to a more unfolded state. Transitions between these states can be fitted by a Hidden Markov model (HMM). (B) Example results after data analysis of constant distance traces of unpublished results. The construct used is OpuAC from van der Sleen et al.^1^ Upon increase of the trap distance (and force), the probability of the unfolded state and unfolding rate increases. Data can be fitted to obtain the energy differences between states and the transition rates at zero applied force. Each point belongs to a constant distance trace plotted in panel (A). Number 1 belongs to the trace with the shortest trap distance (lowest force), whereas number 7 was recorded at the largest trap distance (highest force). The error in probability resulting from the finite measurement was estimated using Monte Carlo simulation.^24^

## Expected outcomes

This protocol should yield oligo-labeled protein in good quantity and quality (500 μL of ∼ 0.1 mg/mL labeled protein). One round of purification and labeling should yield enough aliquots for > 10 measurement days. The “best” fraction from SEC usually still contains a significant fraction of single-labeled species. The double-labeled species should be clearly visible on an agarose gel, however this may depend on the separation capabilities of the SEC column. A trained C-trap user should be able to capture 10 – 20 bead pairs within 2 h, of which the formation of tethers should be observed every 3 bead pairs (although this is highly dependent on the labeling efficiency and the concentrations used). On a measurement day, typically 1 – 5 promising tethers are observed and measured, although after further analysis < 10% of the promising tethers can be used for further data processing (this estimate is also highly sample-dependent). A good tether should be stable for tens of minutes of measurement. Rips in the force-distance data due to protein unfolding are typically observed below 40 pN (but this may be highly specific for the protein of interest), whereas rips corresponding to interactions are usually found in the lower force regime (< 15 pN).

## Limitations

This protocol describes sample preparation and measurement to study ABC transporter OpuA in lipid nanodiscs using smOT. The protocol is generally applicable for other proteins, but there are some limitations. First, a structure (or a reliable structural model) should be available of the protein to design suitable cysteine-mutations and perform analysis on the obtained force-distance data. Additionally, the protein should be stable for several hours at room temperature (∼ 20 °C) and can preferably be stored at −80 °C for several months after labeling with maleimide-oligonucleotides.

The measuring method itself, smOT, also has its limitations due to its single-molecule nature. The throughput of a measurement is low since only a single molecule can be measured at a time. We aim to have ≤ 33% of the caught beads pairs to have successful tether formation, to increase the chance of formation of single tethers rather than two or three tethers being formed simultaneously, which also lowers the throughput. Lastly, reproducibility of sample preparation is very sensitive to small changes since small volumes are pipetted.

## Troubleshooting

### Problem 1

The protein of interest cannot be labeled via cysteine mutations.

### Potential solution

Some proteins contain surface-accessible native-cysteines that cannot be mutated, which may hinder specific labeling by a maleimide-construct. There are alternative labeling strategies, such as a peptide-tag or non-canonical amino acids. For an overview, see also van der Sleen and Tych.^27^

### Problem 2

The purification of a stable membrane protein is not successful (related to Step 2 – 6).

### Potential solution

Membrane proteins are in general more challenging to work with then soluble proteins. There are a few common practices in the lab that may improve the stability. (1) Always have the protein in a cooled environment. (2) Handle the protein gently; do not vortex the sample and avoid as much mechanical agitation as possible, reduce the air-water-interface as much as possible by gently pipetting and finding appropriate tubes for the volumes when agitating. (3) Add 20% (v/v) glycerol to all solutions used for the sample (but note that glycerol may affect the efficiency of reconstitution into nanodiscs). Additionally, the detergent and buffer used for solubilization may greatly affect the stability of the protein and may be specific for the protein that is purified. The detergent (DDM) and the concentrations used for DDM in this protocol are a good start, but it may be beneficial to screen for other detergents and buffers.^28^ The lipid composition for reconstitution may also have an effect on the stability and functionality of the protein, and additionally may affect the reconstitution efficiency.

### Problem 3

The nanodiscs that are formed by reconstitution are not monodisperse and vary in size (related to Steps 4,6).

### Potential solution

A range of different membrane scaffold proteins (MSP) are available.^29^ Larger MSPs are known to form more heterogeneous nanodiscs. The most optimal protein:MSP:lipid ratio is highly dependent on the protein used and the protocol used for the solubilization of lipids. Other factors that play a role are: (1) the concentration at which protein is reconstituted, (2) the final detergent concentration in the reconstitution mixture, (3) the reconstitution time and temperature before and after the addition of BioBeads. An excess of MSP relative to protein is always used to prevent multiple proteins inserting in a single nanodisc.

### Problem 4

The labeling efficiency of the protein by the maleimide-oligonucleotides is low (related to Step 5, 7).

### Potential solution

The labeling efficiency is dependent on the availability of the cysteine position on the protein. First, cysteine mutation should be surface-accessible (and not in the membrane). Additionally, surrounding residues may affect the pKa and therefore the reactivity of the cysteine. It is therefore important that there are several cysteine positions tested, if the first one is not working. The accessibility and reactivity of the cysteine can already be tested with cheaper maleimide-conjugates, such as Mal-PEG. The labeling efficiency of Mal-PEG can be determined by running the sample on an SDS-PAGE gel. Older CMVs (stored longer than a year) lose reactivity of the cysteine, therefore preferably CMVs are used for labeling within a few months.

Once a suitable cysteine position is found, a few alterations can be made to the protocol as described above. Increasing the pH to 7.5 improves reactivity of the cysteine. It is however important to be aware that increasing the pH even further risks that lysine residues may be labeled. Additionally, a larger excess of maleimide-oligonucleotides can be used and/or the reaction volume can be decreased. Finally, incubation time and temperature may be increased, but the effect on protein stability should be taken into account.

The reducing agent is also important. It is essential that DTT is completely removed during the washing step (Step 5D), prior to the addition of the maleimide-oligonucleotides since DTT irreversibly can react with maleimide-groups. As an alternative, tris(2-carboxyethyl)phosphine (TCEP) can be used.

Alterations in the step where the protein is labeled can also be made. It is for instance possible to label the protein in the detergent-solubilized state when bound to the Ni^2+^-Sepharose in Step 3. Alternatively, one can label during reconstitution when using TCEP as a reducing agent and adding the maleimide-oligonucleotide to the reconstitution mixture in Step 4. Make sure to check that the maleimide-oligonucleotide is not non-specifically insert into the nanodisc using a wild-type protein as control, since we have observed non-specific insertion of fluorophores using a similar protocol. Note that higher amounts of maleimide-oligonucleotide are required with these alternatives. It is also possible to first purify the protein with SEC, incubate the protein with maleimide-oligonucleotides in solution and isolate the labeled construct. It is important to make sure that the protein is in sufficiently high concentration when labeling to be detectable in the SEC chromatograms.

### Problem 5

The protein-oligo construct behaves differently after storage at −80 °C, in comparison to when the construct is freshly used.

### Potential solution

Storage at −80 °C may affect protein stability. Addition of glycerol to the protein construct prior storage can increase the stability. Addition of glycerol does affect the attachment of the dsDNA handles. Therefore, make sure to optimize the incubation time with the dsDNA handles in the same buffer as the protein is stored in.

Although only small amounts of protein are required for a measurement (only single molecules are measured at the same time), it is preferred to store the protein at least in the μM range. No, or barely any, protein of low concentration may be observed after storage, likely due to a combination of cold denaturation and sticking of protein to the sides of tubes.

### Problem 6

Air bubbles are observed in the microfluidic chip and/or the static chip (related to Step 9,15).

### Potential solution

Prior to the addition of a sample, it is possible to flush the chip with a low surface tension liquid, *e.g.*, 20% (v/v) ethanol. In the case of the static chip, when adding the first liquid, it is important to add it from the side with the big opening and not from the individual channels to avoid introducing bubbles.

### Problem 7

It is not certain whether one or multiple beads have been caught in a single trap based on visual inspection (related to Step 17).

### Potential solution

Measuring a much higher trap stiffness than expected may indicate that two beads in a single trap have been caught and/or dirt may be attached to the bead. Alternative ways to detect the trapping of multiple beads are: (1) re-catching the bead when there is no flow by shutting the appropriate laser for a short time, and (2) moving the trap quickly around. You may visually observe multiple beads and/or dirt on the bright-field image.

### Problem 8

The amount of tethers observed during a measurement session is not optimal (related to Step 18).

### Potential solution

Each time a new sample is prepared, the incubation conditions may need to be optimized. Once finding the most optimal incubation conditions, also tether formation can be improved by variations during the tethering procedure. If too many tethers have been observed within one sample prep, it is possible to stop the approach curve early (spatially separate the beads more) and/or wait for a shorter time at the distance where the beads are close to each other. Alternatively, when too few tethers are observed, one can wait for a longer time at the position where the beads are touching.

### Problem 9

The beads fall into the other trap and remain stuck to each other when performing the approach curve (related to Step 18).

### Potential solution

Varying the salt concentration may improve the experiment (though it will of course also have an impact on the behavior of the protein). We have observed that at high salt concentrations (600 mM KCl), the beads tend to be more “sticky”. A recommended starting point would be a salt concentration of 200 mM (though this may depend on the salt used). If high salt concentrations cannot be avoided, one could first explore the position of the lasers required to have the beads come into contact. Then, the beads can be shifted to the higher salt concentrations and the tethering can be done tens of nanometers away from the position where the beads are touching.

### Problem 10

The tethers that have been caught are unstable and easily break below 40 pN (related to Step 19).

### Potential solution

To improve stability and avoid radiation damage, it is important to avoid the formation of radical oxygen species. Make sure to remove as much oxygen from the buffer shortly before use. In addition to the proposed sonication in Step 8, the buffer can be flushed with nitrogen gas prior sonication and the sonication can be performed under vacuum. If no sonication setup is available, one can also vigorously stir the buffer while flushing with nitrogen and subsequently pulling a vacuum.

Other alternatives to reduce radiation damage is to decrease the exposure of the protein to the laser. This can be achieved by lowering the laser intensity and/or the use of larger beads.

### Problem 11

Force-distance curves have been recorded, but it is hard to identify to which event (*e.g.*, what is unfolding) each of the rips in force-distance curves can be assigned to (related to Step 21).

### Potential solution

To identify to which event the rips in the force-distance curves can be assigned to, it is essential to note every possible protein-unfolding event and/or breaking of an interaction. In addition to the 3D structure, we recommend the use of the domain organization in 2D which can for instance be found with PDBsum.^30^ This may support the identification of the more mechanical stable regions. Note that it should be taken into account that the pulling direction on β-sheets may affect their mechanical stability. Some proteins may not have clearly distinct mechanically stable domains. It may therefore be beneficial to perform Molecular Dynamics simulations^1^ to identify or predict the unfolding stages. Lastly, it is also possible to develop constructs where the cysteine mutations are in a different position or produce truncated protein constructs to compare the unfolding patterns observed.

### Problem 12

The microfluidics system is not clean or clogged (chip and/or syringes). Particles are observed when flushing with liquids when there should not be any particles or the liquid level is not going down at the same rate in all syringes (related to Step 9 and 14).

### Potential solution

If the system is not clean, several measures can be taken. The disposable syringes can be replaced or other cleaning reagents can be used (e.g., bleach). In case of the use of bleach, make sure to wash away the bleach with MQ and consecutively flush the system with a reducing agent (e.g., 10 mM sodium thiosulfate). Alternatively, an air bubble can be introduced on purpose to remove beads that are stuck on the surface.

If one of the channels is clogged, apply pressure waves on the clogged channel. This is achieved by opening only the clogged channel and the waste channel, and apply a series of pressure changes in the applied pressure. Alternatively, a plunger from another syringe can be used to apply manual pressure on the clogged channel. This can also be done in waves. Make sure to be careful to not break any of the equipment. If the blockage is in the tubing, it is often at the end of the tubing where the connection to the chip is. It is possible to remove some of this tubing (∼ 5 mm) to remove this blockage.

## Resource availability

### Lead contact

Further information and requests for resources and reagents should be directed to and will be fulfilled by the lead contact, Katarzyna Tych (k.m.tych@rug.nl).

### Technical contact

Technical questions on executing this protocol should be directed to and will be answered by the technical contact, Lyan van der Sleen (l.m.van.der.sleen@rug.nl).

### Materials availability

Plasmids generated in this study are available upon request without restrictions.

### Data and code availability

This paper does not report original code. Any additional information required to perform data analysis as described in this protocol is available upon request from the lead contact, Katarzyna Tych (k.m.tych@rug.nl).

## Acknowledgments

We acknowledge funding from the Dutch Research Council: NWO OCENW.KLEIN.526. We thank D. Linnik for his critical feedback on the figures.

## Author contributions

L.v.d.S. and M.v.d.N. purified, reconstituted, and labeled OpuA. L.-M.S. and K.T. helped L.v.d.S. to set up all smOT-related experiments. Data for Figure 2A were provided by L.-M.S. and data for Figure 2B by M.v.d.N. L.v.d.S. wrote the manuscript with the help of K.T. All authors supervised and revised the manuscript.

## Declaration of interests

The authors declare no competing interests.
